# Medical Education and State License Examinations1Presented to the Medical Society of the State of New York, at its ninety-first annual meeting, Albany, January 26-28, 1897.

**Published:** 1897-03

**Authors:** William Warren Potter

**Affiliations:** Buffalo, N. Y., Examiner in obstetrics, New York State Medical Examining and Licensing Board


### Progress in Medical Science.

### MEDICAL EDUCATION AND STATE LICENSE EXAMINATIONS.[1]

CONDUCTED BY WILLIAM WARREN POTTER, M. D., Buffalo, N. Y.,
Examiner in obstetrics, New York State Medical Examining and Licensing Board.

### REPORT OF THE NEW YORK STATE MEDICAL EXAMINING BOARD.

FROM January 1, 1896, to January 1, 1897, there have been 805 candidates for licenses to practise medicine in the state of New York who have appeared before the several boards having the power to recommend the granting of a license to practise medicine. The total percentage of rejections was 23.7. Before the board representing the Medical Society of the State of New York there appeared 733 candidates, of whom 555 were successful and 178 unsuccessful, the percentage of rejections being 24.2. Before the homeopathic board there appeared 52 candidates, of whom 41 were successful and 11 unsuccessful, the percentage of rejections being 21.1. Before the eclectic board there appeared 20 candidates, of whom 18 were successful and two were rejected, or 10 per cent.

There were five examinations during the year—in January, April, May, June and September. In the figures above given, each candidate who appeared more than once during the year is counted for each time he appeared. The figures in the table appended are for individuals regardless of the number of times they appeared for examination. This latter table shows that 86.2 per cent. of graduates of New York medical schools were successful in our examinations, while but 74.5 passed our examinations of those who were graduates of foreign (*i. e.*, out-of-the-state) colleges. The program of examinations for the year 1897 contemplates five examinations at intervals similar to those for 1896.

The differences in the ratings of the various medical colleges of the state are matters of public record and are not here reproduced because of the very little differences in the percentages to be noted. The records are on file in the regents' office, and to those interested the exact figures will be furnished on request.

It was feared by persons interested in the medical institutions of this state that the exactions of the law primarily passed in 1890, and subsequently amended in 1893 and in 1896, would deter foreign students from attending the medical colleges of this state.

1. Presented to the Medical Society of the State of New York, at its ninety-first annual meeting, Albany, January 26–28, 1897.

Sworn reports at the regents' office show that since 1893 there has been an increase of 490, or of nearly 15 per cent., in the number of medical students in New York state. It is also to be noted that at the present time the state of New York furnishes education to about 17 per cent. of all the medical students in the United States. From the years 1889 to 1893 there was an apparent decrease in the number of medical students in the state of New York, presumably because under the old methods of nomenclature students of pharmacy and special courses were included in the list of students of medicine. At the present time this does not obtain. Students of medicine alone, seeking for the degree of doctor of medicine, or post-graduate students, are now counted in the list. Under the highest matriculation requirements in the United States, the state of New York is increasing rapidly in the number of her medical students. The increase of a beneficent character in connection with New York medical schools is not alone confined to the added number of students above noted, but in this connection we call attention to the fact that since 1893 the total property of the medical schools of the state of New York has increased more than 100 per cent. The above figures in connection with the medical colleges of the state of New York are given, because it is believed that after the lapse of six years in the enforcement of the medical laws the objection urged repeatedly against the establishment of the law, which carried with it the statement that the influence and the power of the medical colleges in the state of New York would decrease because of the restrictions placed on admission to our schools, has fallen flat; and per contra, that the prediction of those who warmly advocated and pressed to a final passage the law under which we are now working has been verified—namely, that the highest standard would ultimately redound to the greatest credit of the state. As the growth referred to is not confined to the colleges of New York City, but applies even more to those situated in other parts of the state, it will be seen that the increase has been equally distributed and that no one particular institution, or set of institutions, has been favored by the operations of the law.

The methods of examination which originally obtained in the board have, with slight modifications, been continued. The main subject of medicine is subdivided into seven groups, as specified in the law. To each one of these subdivisions a time limit of three hours on examination is devoted. In this manner the entire session is made to last three and one-half days. In each subdivision there are fifteen questions submitted, of which the candidate must answer ten to be selected by himself. The complete value of each question answered correctly is ten, so that an absolutely perfect paper would receive 100 points. It is necessary in order that the candidate be passed in a topic to receive at least seventy-five points. If he fails in one topic, the board refuses to recommend him for license, and it becomes necessary for him to appear at a subsequent examination, without extra cost if his total average is 80 per cent. or more, in which case he may be reëxamined only in the topic in which he was rejected. Should his total average in the other topics be less than 80 per cent., or should he be rejected in more than one topic, it is required that he wait at least six months before reëxamination, without cost, and in all the topics ; or, if he desires to take an examination sooner than within the time limit here given (and the law is quoted in this part), he may, on payment of an additional fee of $25, be admitted to the examination next succeeding the one at which he was rejected.

It will be seen from these figures that before being licensed to practise medicine an applicant must receive at least 525 points out of a possible 700. The annual report of the State Board of Medical Examiners of a neighboring state is so ridiculous in its pretentions of comparison with the work of our own board that we feel it our duty to detail the methods by which a conclusion is reached by them. They state that the regents refuse to accept their certificates in lieu of further examination for license to practise in the state of New York, "although our medical requirements are higher than theirs, since 675 points must be obtained by a candidate to secure our license and only 525 points to secure theirs." The entire subject of medicine in their state is divided into nine subdivisions. They have accepted our method of marking, and consequently require 75 per cent. in each topic. They multiply 75 by 9 and thus get a total of 675 points out of a possible 900. Ours is, as stated, a total of 525 out of a possible 700. It will be seen from this that the percentage is exactly the same. However, their own figures of the examinations of 1896 will show how little reason they have for the statement that their medical requirements are higher than ours. Notwithstanding our high test for entrance to examination, a condition which does not obtain with them, we rejected last year 23 per cent. of all the candidates who were admitted to examination. Their board rejected just 4 per cent.

Comparisons may readily be made by those who are interested, when the speciousness of the claim will become apparent.

Notwithstanding the legislation of last year, the standard of the board is still maintained. The minor concession which was made for the benefit of those students who had registered under a misapprehension in 1894 and 1895, and which had the approval of almost the entire profession and of all the medical colleges but one in the state, has operated to the credit of our institutions and has reflected favorably on the liberal spirit of the medical educators of the state.

Because of our failure to reciprocate, the State Board of Medical Examiners of Pennsylvania has refused to recognise our license as heretofore, and in consequence practitioners of medicine licensed to practise in New York state are required to pass an examination before being permitted to practise in Pennsylvania. The only reason for this is because of our failure to reciprocate. This we have been unable to do because of the lax methods of admission to examination prevailing in Pennsylvania, as compared with the stringent rules in force in this state. The New York statute makes it impossible for us to show any favor to an out-of-the-state applicant for license which the laws of the state of New York do not extend to an applicant residing in our state. The discrimination would be an unjust one, and in keeping with this statutory provision we are forced to refuse the credentials of licentiates of other boards. We hope that there may be some modification of this rule, but the initiative must come from other states where the regulations as to examination, more particularly those applying to the preliminary or academic requirements, must equal ours. We are fortunately situated in this state in that we have an independent educational body known as the Regents of the University of the State of New York, which supervises the higher education of our state. This body is in possession of all the facts and figures relating to higher education throughout the country and throughout the world, and from its files can be learned whether or not an institution from which an applicant for license is graduated maintains a standard which is equivalent to that obtaining in our own state. No other institution in the world, probably, is so well equipped with information of this character as is the regents' office, and we derive the benefit of their experience. In Pennsylvania and in other states the law simply requires that an educational test be had as to the qualifications of those who desire to enter on the study and practice of medicine. A board is improvised for the conduct of preliminary examinations, but the tests are not uniform. In many states the medical colleges themselves pass upon the qualifications of those who wish to have themselves entered as medical students, and the preliminary tests thereby become a farce. As a matter of consistency, we cannot place our own men, who have passed through the various examination tests prescribed by the regents, to the disadvantage which would certainly be theirs if the lower requirements of other states were to be recognised as equal to ours. We feel, however, that no step can be taken by this board which would in any way indicate that there was any lowering of the high standard which the society has set for it in the years of conflict to create public opinion in its favor. We are satisfied that today the laity, as well as the profession, would not for one moment permit a backward step in medical education or legislation in the state of New York.

From time to time during the year sets of medical question papers are distributed among the members of the medical profession throughout the state and country in the hope that a study of the same will produce results in the way of criticism which will be of value to our board. We are pleased to say that many members of this society are deeply interested in our methods, and that suggestions and criticisms come from them, which are acceptable, because they are made in good faith and are valuable. We trust for a continuance of this interest on the part of the members of this society.

The assignments of the various subdivisions of the realm of medicine as given in the law are as they were originally made in September, 1891, the officers of the board also continuing as originally elected.

Up to January 1, 1897, 1,893 licenses have been issued under the law which established this board. From this it can be readily seen that within a very short period a majority of the practising physicians of the state of New York will have been licensed by the state authorities, in addition to having a diploma from an accredited medical college, subsequent to a thorough preliminary education and a complete training in medicine.

Imperfect registrations were corrected as follows during the year: by the State Board, 18 ; by the Homeopathic Board, 4 ; by the Eclectic Board, 1.

Since the establishment of the system at present obtaining,

2,832 doctors of medicine have applied for the right to practise medicine in our state. Under the old law, each one of these would have been entitled to practise his profession unquestioned, on registering his diploma at a county clerk's office. Of these, 927, or 29.2 per cent., were found incompetent and deficient. A system which has saved the state from numbering among its legal practitioners of medicine so large a percentage of persons whose presence as such must surely have worked harm to the community, and must have detracted from the worth and dignity of our profession, needs no encomiums at our hands.

Signed,

William C. Wey, *President*.

Maurice J. Lewi, *Secretary*.

William S. Ely.

George Ryerson Fowler.

William Warren Potter.

J. P. Creveling.

Eugene Beach.

---

THE PENNSYLVANIA STATE BOARD OF MEDICAL EXAMINERS.

The rapidly increasing standard (*Med. News*) required by this board is an encouraging indication of progress toward the general elevation of scientific medicine in America. Nearly 38 per cent. of the applicants were rejected by the board at its December meeting, while the percentage of failures in July last was only fifteen. Bacteriology is said to have been the subject upon which many showed an insufficient knowledge. The scope of each examination is intentionally broadened, and a more varied as well as a more precise acquaintance with all departments relating to practical medicine will be exacted at each succeeding session of examiners. While sympathy might be felt for those who fail, congratulation for the worthy should be extended by all who have the glory and honor of the profession at heart.

---

*The Session of the Board of Medical Examiners in December.* —At the last session of the Board of Medical Examiners, representing the Medical Society of the State of Pennsylvania, held December 14th to 17th inclusive, there were eighty-eight applicants for license to practise. Of this number five were women and eighty-three men. The result of the examination showed twenty-nine failures and one expulsion, on the first day of the session, for copying. It will thus be seen that much inferior medical timber is kept outside the boundaries of the state, to the great advantage of its citizens and to the greater glory of the medical profession. The next session of the board will probably be held in June at Harrisburg.—*Pittsburg Med. Review.*

---

PRELIMINARY VS. FINAL REQUIREMENTS.

THE step taken by a number of the state boards of health in regard to preliminary requirements for admission to medical colleges is in the right direction, but it is a question if it is not too far in advance of the requirements for graduation. A diploma from a literary college does not always signify ability, neither does a diploma from a medical college always mean that the graduate is competent to practise the art. Those states which insist upon the student of medicine being so thoroughly equipped from a literary standpoint might be a little more careful in the qualifications which they require for practising. Something more than a knowledge of grammar, arithmetic and history is required of a doctor of medicine ; something more than the fact that he has passed through the curriculum of a high school or college is necessary to prove his adaptability to the work of medicine. Something more than the ability to memorise text-books and lectures is necessary to the man who expects to put into every-day practice the teachings of a life-saving art. A diploma from a high school or college is far from being a qualification for the study of medicine. The applicant for entrance to a medical college should be able to prove his qualification by a careful examination upon those subjects most necessary for the study of such a vast science. But shall we stop with the qualifications for the study of medicine ? The most important feature in this wave of restriction should be rather at the latter end of the course. Hundreds of students are graduated each year who have not the least idea of the practical application of the principles they have been taught. Let us have examining boards who will examine the output of our medical colleges from a practical standpoint. Let us have them put to the test in the hospital and at the bedside. Few men in the profession can have confidence in the sincerity of the motives for some of the rulings which have recently been made, for otherwise they are libels upon the judgment and foresight of the men who have been placed at the head of state medical affairs. — *Editorial*, *Kansas Medical Journal.*

